# An improved method for high-temperature induced embryo sac chromosome doubling in *Populus simonii* Carr. × *P*. *nigra* var. *italica* (Moench.) Kochne, an interspecific hybrid of *Tacamahaca* and *Aigeiros* poplars

**DOI:** 10.1038/s41598-018-37297-w

**Published:** 2019-01-24

**Authors:** Wenting Xu, Liqin Guo, Yan Zhang, Jian Zhao, Zunzheng Wei, Jinfeng Zhang

**Affiliations:** 1 Beijing Advanced Innovation Center for Tree Breeding by Molecular Design, National Engineering Laboratory for Tree Breeding, Key Laboratory of Genetics and Breeding in Forest Trees and Ornamental Plants of Ministry of Education, Key Laboratory of Forest Trees and Ornamental Plants biological engineering of State Forestry Administration, College of Biological Sciences and Technology, Beijing Forestry University, Beijing, 100083 P. R. China; 2 State Key Laboratory of Subtropical Silviculture, School of Forestry and Biotechnology, Zhejiang A&F University, Hangzhou, 311300 P. R. China; 3 Beijing Academy of Forestry and Pomology Sciences, Beijing, 100093 P. R. China; 4 Beijing Vegetable Research Center, Beijing Academy of Agriculture and Forestry Sciences, Key Laboratory of Biology and Genetic Improvement of Horticultural Crops (North China), Key Laboratory of Urban Agriculture (North), Ministry of Agriculture, Beijing, 100091 P. R. China

## Abstract

Chromosome doubling is considered an important technique in poplar breeding, with many triploid clones being artificially induced and selected for promotion in the north and northeast of China because of their outstanding traits in vegetative growth and environmental adaption. In this study, the triploid yield of *Populus simonii* Carr × *P*. *nigra* var. *italica* (Moench.) Kochne was 23.41%, which exceeded the yield attained in our previous studies due to the use of an optimized method of chromosome doubling in the embryo sac at a high temperature. The development of the embryo sac after the pollination of this hybrid was investigated to determine the induction period. Ploidy of seedlings was identified by flow cytometry after initial filtering using the chloroplast counting method. Eleven triploids and one tetraploid were ultimately obtained, and the optimal operating conditions were exposure of female catkins to 41 °C for 2 h at 66 h after pollination (HAP). This study identified an efficient method of chromosome doubling in *P*. *simonii* × *P*. *nigra* var. *italica* and provided several polyploids for *Populus* polyploid breeding programs and subsequent studies.

## Introduction

The induction of chromosome doubling during hybridization is an important strategy in the polyploid breeding of *Populus*. Three different approaches to the procedure vary according to the object treated: (1) 2n pollen induction, (2) 2n female gamete induction, and (3) zygotic embryo chromosome doubling. Compared to haploid pollen, 2n pollen exhibits developmental retardation of the pollen tube during fertilisation, which makes triploid induction inefficient^1,2^. In addition to its weak competitive ability against haploid pollen, the increased paternal genome from 2n pollen disrupts the parental genome balance, which may lead to overproliferated endosperm and seed abortion^3^. Female catkins are used as the treatment materials in zygotic embryo chromosome doubling and 2n female gamete induction. These approaches are more efficient than 2n pollen induction due to the avoidance of pollen competition. Uniparental maternal plastid inheritance is dominant in angiosperms^4^, which means that triploids originating from the female parent would inherit more genetic information than triploids originating from male parent. Transcriptome sequencing in *Spartina* has suggested that allopolyploidization is accompanied by maternal expression dominance^5^. Both the megaspore (meiotic doubling) and embryo sac (mitotic doubling) could be induced at different stages of megagametogenesis. Female catkins during certain developmental stages before or after pollination have been treated with colchicine, and triploids from 2n female gametes were obtained^6–8^. In contrast, high temperature, as a physical mutagenic agent, is more effective for the induction of polyploids due to reduced injury, coincident treatments, and operational advantages. This technology was first applied to sect. *Leuce*, and high induction rates were achieved. In *P*. *adenopoda*, female gametes were treated with high temperature during the megasporogenesis and embryo sac stages, and the triploid induction rates were 40 and 83.3%, respectively^9^. The average triploid induction rate was 62.06% in *P*. *tomentosa* when dealing with the embryo sac^10^. In *P*. *alba* × *P*. *glandulosa*, the highest efficiency of triploid induction was 87.0% for the embryo sac^11^. These studies obtained higher induction rates than did previous studies using male gametes. Hybrids between sect. *Tacamahaca* and sect. *Aigeiros* were also used for embryo sac induction at high temperature. During the embryo sac developmental stage, *P*. *pseudo-simonii* × *P*. *nigra* ‘Zheyin’ was treated with high temperature, and the triploid induction rate was 40%^12^.

Hybrids between sect. *Tacamahaca* and sect. *Aigeiros* are especially significant for forestation in the northeast of China because of their excellent stress resistance and growth characteristics. Our research group has focused on chromosome doubling during hybridization between the two sections for several years and has established a technical system for high-temperature polyploidy induction^13^. The development of the female gamete and zygotic embryo in *P*. *simonii* was observed to confirm the time required for the induction treatment, and cross compatibility was used to determine the parents and cross combinations^13^. Finally, we chose *P*. *simonii* and *P*. *simonii* × *P*. *nigra* var. *italica* as female parents and *P*. *simonii* × (*P*. *pyramidalis + Salix matsudana*) and one of its clones as male parents. According to the cytological result, chromosome doubling in the high-temperature hybridization between sect. *Tacamahaca* and sect. *Aigeiros* was implemented, and polyploidy offspring were obtained in several cross combinations. The triploid yield of *P*. *simonii* × *P*. *nigra* var. *italica* was 11.76% using *P*. *simonii* × (*P*. *pyramidalis* + *Salix matsudana*) as the male parent. The most efficient combination was a 38 °C treatment for 4 h at 72 h after pollination (HAP).

*Populus simonii* × *P*. *nigra* var. *italica* is a female interspecific hybrid between sect. *Tacamahaca* and sect. *Aigeiros*. It is a major cultivated variety that has been generalised in a broad region of northeast China due to its outstanding stress resistance and environmental adaptability under desertification conditions and with improvement of the ecological environment. The pollen donor (*P*. *simonii* × (*P*. *pyramidalis* + *Salix matsudana*) clone) was selected due to its hybridization compatibility with the female parent^14^, complementary advantages in growth vigour, and its straight stem. *Populus simonii* × (*P*. *pyramidalis* + *Salix matsudana*), a hybrid between sect. *Tacamahaca* and sect. *Aigeiros*, has been widely used for improvement of *Populus* breeding, with many derivative hybrids used in northeast China due to their outstanding traits in satisfying local breeding objectives. In conclusion, the induction system established in our previous study could be used to produce excellent polyploidy clones using *P*. *simonii* × *P*. *nigra* var. *italica* and *P*. *simonii* × (*P*. *pyramidalis* + *Salix matsudana*) clones as their parents.

In this study, a high-temperature induction treatment applied after pollination was studied for the cross-combination *P*. *simonii* × *P*. *nigra* var. *italica* × *P*. *simonii* × (*P*. *pyramidalis* + *Salix matsudana*) clone. However, no polyploidy seedlings were produced from the induction experiment, which was performed under the conditions employed in our previous research. The probable cause of this was imprecise judgment of the embryo sac developmental stages in the female parent. In consideration of this, we first investigated the developmental process of the embryo sac after pollination by evaluating paraffin sections. Finally, we obtained a certain amount of polyploidy seedlings and optimized the high-temperature induction treatment conditions according to the statistical analysis of an orthogonal experimental design after a preliminary experiment to assess tolerance to high temperatures.

## Results

### Cytological observation of megasporogenesis and megagametogenesis

**Figure 1:**
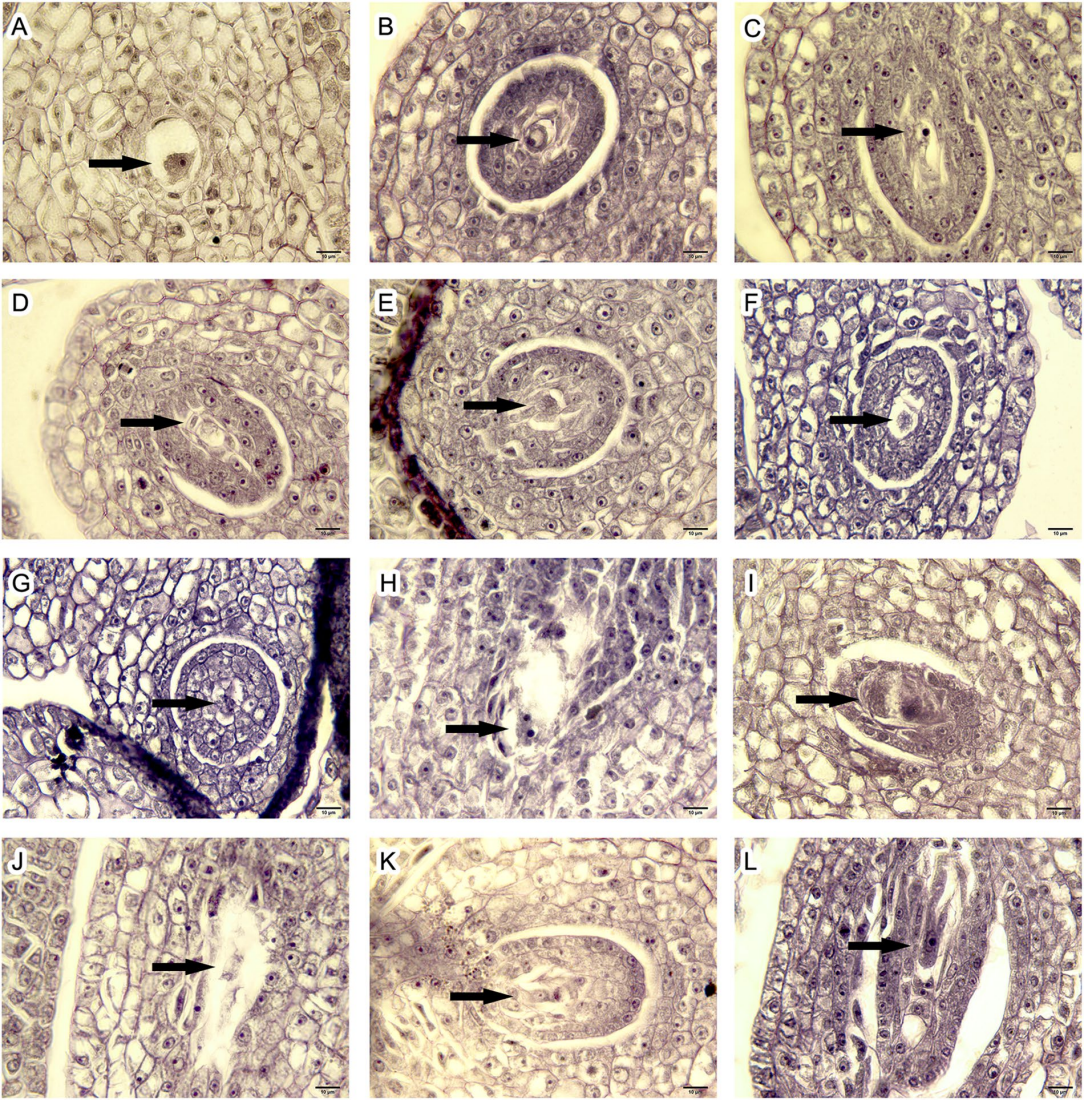
Megasporocyte meiotic stages in *Populus simonii* × *P*. *nigra* var. *italica* (bar = 10 μm). (**A**) Megaspore mother cell; (**B**) Leptotene; (**C**) Pachytene; (**D**) Diakinesis; (**E**) Metaphase I; (**F**) Anaphase I; (**G**) Telophase I; (**H**) Prophase II; (**I**) Metaphase II; (**J**) Anaphase II; (**K**) Telophase II; (**L**) Linear tetrad.

The developmental model of an embryo sac in *P*. *simonii* × *P*. *nigra* var. *italica* was classified as the *Polygonum* type, which could be divided into two stages: megasporogenesis and megagametogenesis. In megasporogenesis, the female archesporium differentiates into megasporocytes, which form a functional megaspore after two cell divisions and one chromosome replication in the meiotic stage (Fig. 1). The functional megaspore was generated from the megaspore located at the chalazal end (farthest from the micropyle), and the other three were arranged linearly and degenerated (Fig. 1L).

**Figure 2:**
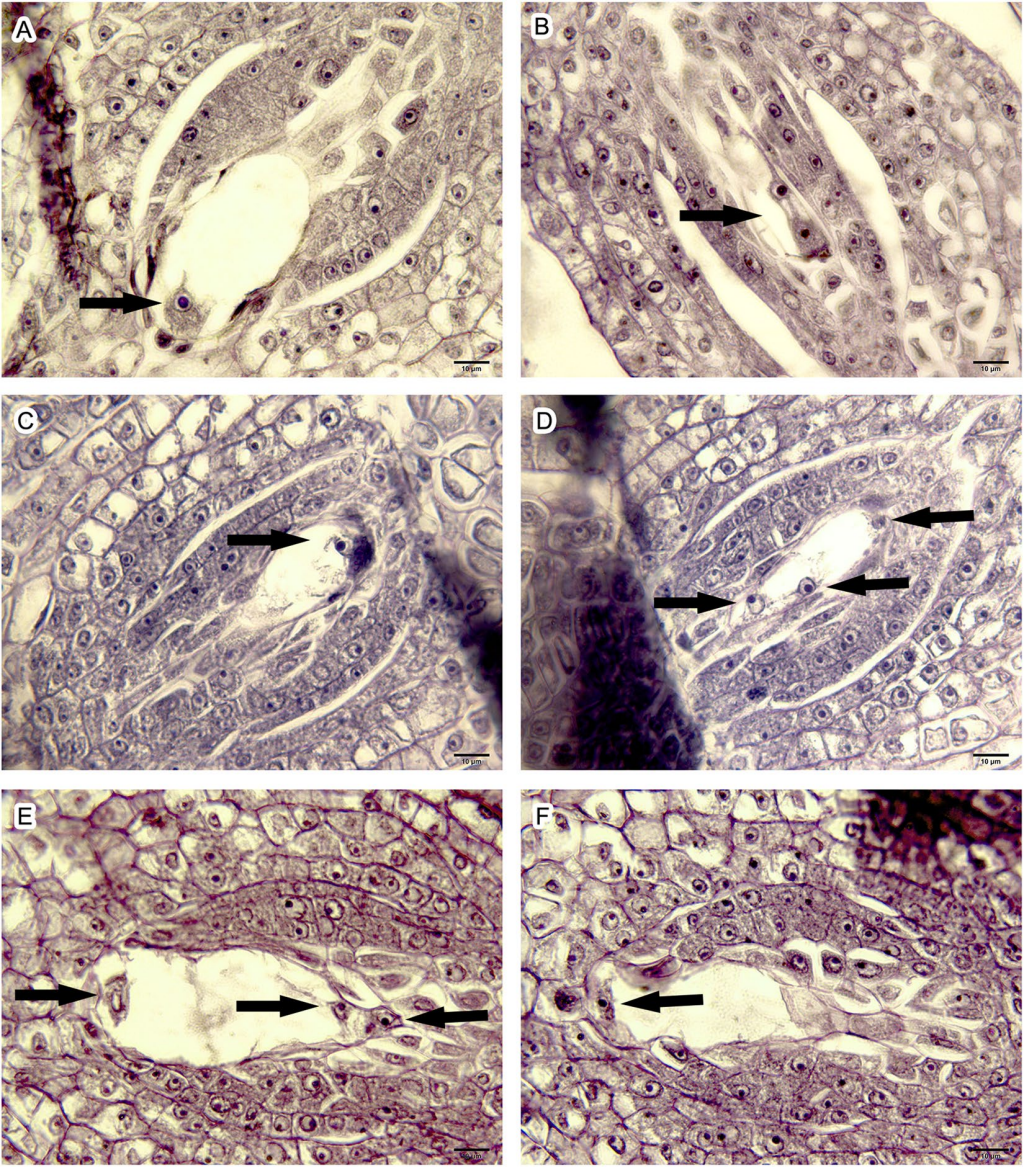
Developmental stages of the embryo sac in *Populus simonii* × *P*. *nigra* var. *italica* (bar = 10 μm). (**A**) A uni-nucleate embryo sac; (**B**) A two-nucleate embryo sac; (**C**,**D**) A four-nucleate embryo sac (serial section); (**E**,**F**) An eight-nucleate embryo sac (serial section).

In megagametogenesis, the functional megaspore located at the distal end from the micropyle developed continually, while the other three cells degenerated and disappeared. With maturation of the functional megaspore, the volume of the cell increased, and a vacuole was present, indicating the beginning of the embryo sac developmental stage (Fig. 2). The megaspore in this stage underwent three mitoses, which were targeted for chromosome doubling at a high temperature and finally formed an embryo sac with seven cells and eight nuclei.

### Statistical analysis of megasporocyte and embryo sac development

Based on the paraffin sections, the development of megasporocytes and embryo sacs displayed obvious asynchrony after pollination, similar to the development of microsporocytes in male catkins^15^. For most of the sampling time (80%), more than four developmental stages were observed, and asynchrony was more distinct in the division stage (Table 1 and Table 2), which provided a longer period of experimental operation for artificial polyploid induction.

**Table 1:** Developmental process of megasporocytes after pollination in *P*. *simonii* × *P*. *nigra* var. *italic*.

Time after pollination (h)	Embryo sac percentage in each developmental stage (%)							^*^Percentage of inducible embryo sacs (%)
	Meiosis I	Meiosis II	Uni-nucleate embryo sac	Two-nucleate embryo sac	Four-nucleate embryo sac	Eight-nucleate embryo sac	Mature embryo sac and post	
0	12.70	28.57	36.51	14.29	7.94			58.73
12	5.00	33.33	35.00	18.33	6.67	1.67		60.00
24	2.94	32.35	35.29	19.12	8.82	1.47		63.24
36	3.17	19.05	36.51	25.40	11.11	4.76		73.02
48		15.15	30.30	31.82	13.64	6.06	3.03	75.76
60			26.87	34.33	22.39	10.45	5.97	83.58
72			16.42	32.84	17.91	19.40	13.43	67.16
96			5.08	20.34	22.03	28.81	23.73	47.46
120					18.03	44.26	37.70	18.03
144						39.62	60.38	0.00

**Table 2:** Capsule survival rate of high temperature preliminary experiment.

Treatment duration(h)	Capsule survival rate (%)			
	36 °C	41 °C	46 °C	CK
1	87.61	90.76	3.05	88.14
3	90.37	48.31	0	
5	86.27	0	0	

As shown in Table 1, over 40% of megasporocytes were still in the meiotic stage when the catkins were undergoing pollination, and most of megasporocytes (36.51%) entering the embryo sac stage were uni-nucleate embryo sacs (Fig. 2A). No eight-nucleate embryo sacs (Fig. 2E,F) or mature embryo sacs were observed. At 12 HAP, the proportion of megasporocytes in meiosis I (Fig. 1B–G) was reduced rapidly from 12.7% to 5%, while the proportion of megasporocytes in meiosis II (Fig. 1H–L) rose by approximately 5%, and eight-nucleate embryo sacs began to appear. Megasporocytes in the meiotic stage were reduced sequentially at 24 HAP, and the percentages in each embryo sac stage were changed slightly. At 36 HAP, the proportion of megasporocytes in the meiotic stage declined sharply once again, from 35.29% to 22.22%, while more megasporocytes developed into the embryo sac stage; the fastest-growing stage was the two-nucleate embryo sac stage (Fig. 2B), in which the proportion of megasporocytes increased from 19.12% to 25.40%. At 48 HAP, meiosis was nearing completion, and only 15.15% of megasporocytes were in meiosis II. The embryo sac developmental stage entered a thriving phase at this time; most embryo sacs were in the uni- and two-nucleate stages, and mature embryo sacs were observed for the first time. Meiosis was finished completely at 60 HAP, and the proportion of megasporocytes in the inducible stage reached a peak. Most (83.58%) of the embryo sacs were in the uni-, two-, and four-nucleate stages (Fig. 2C,D), during which mitosis of the embryo sacs was in progress and artificial polyploid induction could be implemented. At 72 HAP, the development of embryo sacs proceeded, with the proportion in the uni-nucleate embryo sac stage declining to about 10%, while the proportion of embryo sacs that had finished mitosis (eight-nucleate and post-mitosis embryo sacs) increased by 16.42%. The proportion in the inducible stage declined rapidly, from 67.16% to 47.46%, at 96 HAP, and over 50% of embryo sacs had finished mitosis. At 120 HAP, all of the embryo sacs had completed the first two mitoses, and the proportion in the four-nucleate stage (the last remaining mitosis) was only 18.03%. The mitosis of embryo sacs was complete at 144 HAP, with all embryo sacs having achieved the eight-nucleate and post-mitosis stages.

In conclusion, the development of a megasporocyte in *P*. *simonii* × *P*. *nigra* var. *italica* was completed at 60 HAP, and the mitosis of the embryo sac during megagametogenesis was completed at 144 HAP. Thus, to avoid interference from megasporogenesis and obtain high efficiency, the artificial polyploid induction treatment would be best performed at 60, 66, and 72 HAP.

### Preliminary experiment to determine high temperature tolerance

To test the tolerance of capsules to high temperatures, a preliminary experiment was conducted, which showed that about half of the capsules were exfoliated or wizened when treated at 41 °C for 3 h. There was little difference in capsule survival rate under the 36 °C treatment compared with the control, whereas only 3.05% of capsules survived after a 46 °C treatment for 1 h. No capsules survived under treatments of longer duration. These results indicated that the treatment temperature and duration should be reduced.

### Ploidy identification of hybrid offspring and optimization of embryo sac chromosome doubling at a high temperature

After the high-temperature treatment, a total of 467 seeds were harvested, and 388 seedlings were germinated. In the control group were 126 seeds, which generated 107 seedlings. The germination rate of the treatment group was 83.08%, which was approximately the same as that of the control group (84.92%). The chloroplast counting method and flow cytometry were used for ploidy identification.

**Figure 3:**
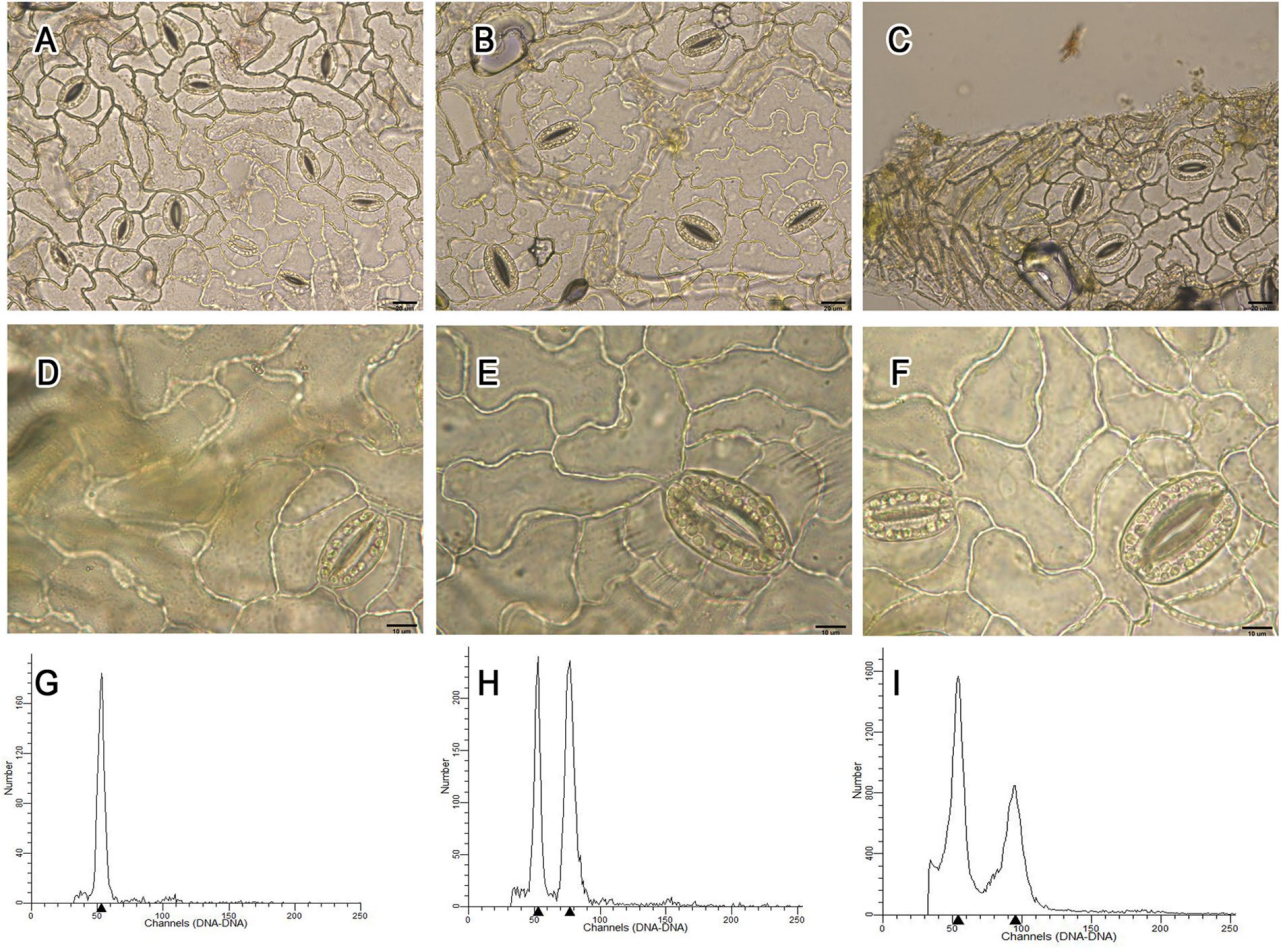
Ploidy identification (The bars in A–C are 20 μm; the bars in D–F are 10 μm). (**A**,**D**) diploids; (**B**,**E**) triploids; (**C**,**F**) tetraploids; (**G**) diploid; (**H**) diploid (left peak) and triploid; (**I**) diploid (left peak) and tetraploid.

Cytological observation showed that the density, size, and chloroplast number of stomatal guard cells differed between diploids and polyploids, whereas there was no obvious difference between triploids and tetraploids (Fig. 3A–F). Stomatal guard cells in polyploid offspring were bigger and contained more chloroplasts (over 13 in one stomatal guard cell and in some cases even over 20) (Fig. 3D–F) than their diploid full sibs, but the density was lower, which meant the number of stomatal guard cells per unit area was less in polyploid offspring than in diploid offspring (Fig. 3A–C).

Flow cytometric analysis was performed to determine the ploidy of seedlings filtered by cytological observation. A nuclear suspension of diploids and polyploids was mixed before measurement to detect the relative DNA content of different ploidy. The results suggested that the DNA content of triploids and tetraploids was 1.5 times and twice that of diploids, respectively (Fig. 3G–I).

A high-temperature treatment was performed following an orthogonal experimental design L_9_ (3^4^); the results are shown in Table 3. A total of 127 candidate polyploids were screened by the chloroplast counting method, with 11 triploids and 1 tetraploid finally confirmed by flow cytometric analysis from one treatment in which catkins were exposed to 41 °C for 2 h at 66 HAP. No polyploids were detected in the control group. The frequencies of triploid and tetraploid induction were 23.4% and 2.1%, respectively.

**Table 3:** High temperature induction of embryo sac after pollination in *P*. *simonii* × *P*. *nigra* var. *italic*.

	Time after pollination (h)	Temperature (°C)	Treatment duration (h)	No. of seeds	No. of seedlings	No. of triploids	No. of tetraploids
1	60	38	4	96	77	0	0
2	60	41	3	22	17	0	0
3	60	44	2	5	0	0	0
4	66	38	3	132	104	0	0
5	66	41	2	53	47	11	1
6	66	44	4	0	0	0	0
7	72	38	2	155	143	0	0
8	72	41	4	0	0	0	0
9	72	44	3	4	0	0	0

As shown in Table 3, the number of seedlings varied among the different treatments. Tests Nos. 1, 4, and 7 had the most seedlings of all treatments, with totals of 77, 104, and 143, respectively. In test No. 5, there were 47 seedlings, including 11 triploids and a tetraploid. Test No. 2 harvested 17 seedlings, but there were no polyploids in this treatment. There were no seedlings in the remaining tests. These results suggest that more seedlings, but no polyploids, were harvested in treatments with a combination of a lower temperature and shorter duration, whereas polyploids were present in the treatments where fewer seedlings were harvested. This meant that weak treatment conditions had less influence on the seedling yield, as well as the mitosis of the embryo sac, which led to a reduction in the induction efficiency. Therefore, the variation in the seedling numbers among the different treatments indirectly reflected the influence of the treatment factors on chromosome doubling efficiency.

High temperature exposure had an evident influence on the development of catkins that made stigma dehydrated and brown and capsules kraurotic, blackened, and even exfoliated. In some treatments, all capsules became kraurotic and contained no mature seeds. A range analysis (Table 4) and variance analysis (Table 5) were conducted to determine the relationship between each of the three experimental factors tested and the acquisition of seedlings.

**Table 4:** Range analysis of seedling numbers.

	Time after pollination (h)	Temperature (°C)	Treatment duration (h)
K_1_	94.00	324.00	77.00
K_2_	151.00	64.00	121.00
K_3_	143.00	0.00	190.00
x_1_	31.33	108.00	25.67
x_2_	50.33	21.33	40.33
x_3_	47.67	0.00	63.33
R	19.00	108.00	37.67

**Table 5:** Variance analysis of seedling numbers.

sources of variation	DOF	SS	MS	F	F_0.05_
Time after pollination(h)	2	634.89	317.44	1.18	19
Temperature(°C)	2	19630.22	9815.11	36.56*	
Treatment duration(h)	2	2162.89	1081.44	4.03	
error	2	536.89	268.44		
total	8	22964.89			

According to the statistical analysis, shown in Tables 4 and 5, treatment temperature was the most influential factor among the three factors tested, with a significantly different seedling acquisition at the three different temperatures. The range analysis revealed that the treatment temperature had greatest influence on the acquisition of seedlings, with the second most influential factor being treatment duration. The developmental stage of the catkins (time after pollination) was the least influential factor on seed and seedling acquisition. In the variance analysis, the F-values for the treatment temperature were 98.31 and 36.56, both of which were greater than F_0.05_, indicating that the three different treatment temperatures used in the test had a significantly different influence on the acquisition of seedlings. The other two factors tested had no significantly different influence on the acquisition of seedlings at all three temperatures.

## Discussion

High temperatures can make microtubules depolymerise by destroying the hydrogen bonds among tubulins^16^, resulting in the absence of spindle during cell division and subsequent doubling of chromosomes. This meant that only during the division stage could cells be influenced by high temperatures, which made the selection of treatment period crucial for chromosome doubling. The development of the embryo sac differed significantly among the varieties of *Populus*, indicating that cytological observation of embryo sacs before induction treatment was necessary. In the sect. *Leuce* embryo sac, three mitoses were completed at about 72 HAP, while the embryo sac in sect. *Tacamahaca* and sect. *Aigeiros* had a hysteretic developmental process, which entered the eight-nucleate and post-mitosis stages at about 144 HAP^6,9–12,17^. Furthermore, different species in the same section had different embryo sac developmental processes. For example, sect. *Leuce*, *P*. *alba* × *P*. *tomentosa* had completely entered the mature embryo sac stage at 60 HAP^6^, whereas *P*. *alba* × *P*. *glandulosa* and *P*. *tomentosa* × *P*. *balleana* entered the same stage at 72 HAP^11^. *Populus euramericana ‘Guariento’*, a hybrid of sect. *Aigeiros*, finished its embryo sac development at 120 HAP^8^, whereas *P*. *simonii*, as a protospecies of sect. *Tacamahaca*, completed its embryo sac development at 144 HAP^13^. In *Populus simonii* × *P*. *nigra* var. *italica*, a hybrid between sect. *Tacamahaca* and *Aigeiros* that was investigated in this study, the embryo sac development process was similar to that of *P*. *simonii*. However, the active mitotic period of the embryo sac was earlier in *P*. *simonii* × *P*. *nigra* var. *italica* than in *P*. *simonii*. The developmental variety among *Populus* species led to differences in the induction period of chromosome doubling in the embryo sac, so observation of the embryo sac developmental process before induction was necessary.

Polyploids were obtained from female catkins of *P*. *simonii* × *P*. *nigra* var. *italica* treated at 38 °C for 4 h or at 41 °C for 2 h, indicating that both low temperature with long treatment duration and high temperature with short treatment duration were suitable treatment conditions for embryo sac chromosome doubling in *P*. *simonii* × *P*. *nigra* var. *italica*. However, the induction rate of a low temperature, long duration treatment (11.76%) was lower than that of a high temperature, short duration treatment (23.4%). Thus, the embryo sac chromosome doubling strategy could be summarised as follows: a long treatment duration at low temperature could be applied to plant materials in which the exact developmental process is uncertain to overlap the most inducible stage and reduce injury, and a short treatment duration at high temperature is more appropriate for induction after confirmation of the developmental process to further improve the efficiency of chromosome doubling.

Previously, a treatment at 72 HAP using *P*. *simonii* × *P*. *nigra* var. *italica* as the female parent produced polyploids^13^, whereas there were no polyploids for the same treatment time in this study. One potential reason for this discrepancy is that the proportion of cells in the inducible stage was reduced at 72 HAP from over 80% to about 67%. Another possible reason for this result was the discrepancy in the judgement of the stigma receptive period among different studies. In *Populus*, the stigma receptive period continues for 1–4 d^18–20^, and the most receptive period could last for several hours. The judgement of the best receptive period in an actual pollination operation depends on indicators such as stigma colour, opening angle, and mucus secretion, and carries the potential for large errors (up to several hours) made by different observers.

The efficiency of high-temperature embryo sac chromosome doubling in *Populus* was significantly different among sections. In sect. *Leuce*, embryo sac chromosome doubling was very efficient, and the triploid yield often greater than 80%. The triploid induction rate of *P*. *adenopoda* and *P*. *alba* × *P*. *glandulosa* was 83.33% and 87%, respectively, while *P*. *tomentosa* had a reported 87.5–100% triploid induction rate in a previous study. In sect. *Tacamahaca* and *Aigeiros*, the highest triploid induction rate was in *P*. *pseudo-simonii* × *P*. *nigra* ‘Zheyin3#’ at 66.7%. In addition, the number of triploid seedlings obtained in an induction experiment in sect. *Leuce* was greater than that in sect. *Tacamahaca* and *Aigeiros*. These results indicated that the response of cell division to high temperatures was species specific, with species in sect. *Leuce* being more sensitive to a high-temperature treatment. The triploid induction rate in this study was 23.4%, which was higher than that in our previous study (11.76%), but still lower than that in *P*. *pseudo-simonii* × *P*. *nigra* ‘Zheyin3#,’ suggesting that cell division in *P*. *simonii* × *P*. *nigra* var. *italica* in our study was less sensitive to a high-temperature treatment.

The superiority of allopolyploidy hybrids in terms of stress resistance and growth vigour in comparison with their diploid parents and full sibs has been generally recognized^21–25^. Technological and conceptual breakthroughs in recent years have provided a novel analytical perspective on polyploidization in genome evolution. Many genomic sequencing analyses in the plant kingdom have revealed that polyploids are prevalent in nature due to their evolutionary advantages for adaption and development^26^. Chromosome doubling during hybridization can achieve allopolyploids, which contain the effects of both allopolyploidization and hybridization in one genome. A substantial reconciliation of incompatibility is required for the newly formed individuals to recover from the “genomic shock”^27^ brought on by both allopolyploidization and hybridization. After the immediate and long-term responses, neopolyploid species experienced multiple cycles of diploidization and chromosomes behaved as normal diploid chromosomes during cell division, while there was no evidence of polyploidization. Thus, an immediate response and some other early regulatory mechanisms after chromosome doubling were difficult to detect in contemporary descendants, which made the neopolyploid material system pivotal. The novel polyploid material system developed in this study can be used for both *Populus* breeding and genome evolution studies.

## Materials and Methods

### Plant materials

Healthy branches with plump flower buds of a female parent *P*. *simonii* × *P*. *nigra* var. *italica* (2n = 2x = 38) and a male parent *P*. *simonii* × (*P*. *pyramidalis* + *Salix matsudana*) (2n = 2x = 38) were collected from plantations in Tongliao City, Inner Mongolia Autonomous Region, People’s Republic of China. These branches were cut into lengths of 1–1.5 m, packed in plastic bags, and transported to Beijing Forestry University. Male parent branches were hydroponically cultured in a greenhouse (10–25 °C) to harvest enough pollen for hybridization before female parent branches were cultured in water. The water was changed every 3 days during the hydroponic process. Pollination was performed with brushes when female caktins entered the receptive period.

### Determination of the developmental stages of megaspore mother cells (MMCs)

Female catkins after pollination were sampled every 12 h for the first 72 h, and then every 24 h until 144 HAP. At least five catkins were collected randomly to ensure that over 50 ovaries were observed in each specimen, enabling a robust statistical analysis. Formalin–acetic acid–alcohol (FAA: 70% ethanol: acetic acid: 40% formaldehyde, 18:1:1) was applied to fix the catkins at 4 °C for 24 h. The sampled catkins were placed in 70% ethanol for long-term preservation. Fixed catkins were dehydrated with a graded ethanol series, and every capsule was embedded in paraffin. After being sectioned with a rotary microtome at 4–6 μm, the trimmed capsules were stained with 0.5% hematoxylin and observed under a light microscope (BX43 Olympus, Japan). Micrographs were taken with an Olympus DP73 camera system.

### Optimization of the high-temperature treatment

Branches with pollinated catkins were removed to a phytotron for a preliminary experiment to investigate temperature tolerance at 36, 41 and 46 °C for 1, 3, and 5 h. At least 100 capsules were sampled on three catkins in every treatment. Based on the results of cytological observation and the preliminary experiment, an induction test was performed at temperatures of 38, 41, and 44 °C for 2, 3, and 4 h at 60, 66, and 72 HAP following an orthogonal experimental design L_9_ (3^4^). Every treatment contained at least five catkins, which were covered with a nylon mesh bag. Untreated branches were defined as a control group. Hydroponic culturing of the branches was continued until seeds were harvested, with the water changed every 3 days. Catkins remained on the branches after pollination for about 35 days before capsule dehiscence and seed release. Dehiscent infructescences in every treatment were collected, and the seeds were removed and stored in vials with a silica-gel desiccant. For sowing, culture traies were filled with sterile nutrient soil (soil: vermiculite: peat, 5:1:1, v/v) in a phytotron under a 16/8-h (light/dark) photoperiod and with 25/20 °C (light/dark) temperature cycling. Seedlings were watered every 2 days.

### Ploidy analysis by stomatal observation and flow cytometry

Huge stomata are a typical characteristic of polyploidy plants^28–30^, and the number of chloroplasts in a stomatal guard cell has been used to identify ploidy in many plant species^28,30–32^. In *Populus*, an abaxial leaf surface was sampled for the observation of stomatal guard cells, which had minimal impact on the growth of seedlings and enabled numerous samples to be screened. This method had previously been applied to sect. *Tacamahaca* and sect. *Leuce*^8,9,33,34^. However, the number of chloroplasts in a stomatal guard cell was not a sufficient condition for identification. The proportion of false positives obtained using this method was high. Therefore, flow cytometry was used for final identification, which contributed to the high detection accuracy and convenient operation of the method compared with chromosome counting.

When seedlings unfolded, with 4–5 euphylla emerging, stomatal guard cells at the abaxial leaf surface were examined for initial ploidy screening. The lower epidermis of the second or third euphylla from the top of the seedling was peeled off and placed into a drop of distilled water on a glass slide, and then observed under an Olympus BX43 microscope. Seedlings with a chloroplast number over 12 in one stomatal guard cell were removed to nutritive cups for subsequent flow cytometry.

Flow cytometry was used to determine the ploidy level of seedlings screened by stomatal observation following Doležel^35^. About 0.5 g of entirely expanding tender leaves of each sample were placed in a petri dish containing 2 ml of extraction buffer and chopped with a sharp razor for 30 s to release cell nuclei. The filtrate, in the form of a turbid liquid that passed through a 50-μm nylon filter, was centrifuged at 800 rpm for 5 min to precipitate the nuclei, which were resuspended with 1 ml of propidium iodide (PI, 50 μg/ml) staining solution with Rnase A and left to stand in darkness for 30 min. Stained samples were detected by a flow cytometer (BD FACSCalibur™, BD Biosciences, USA), and a leaf sample from a known diploid plant (*P*. *nigra*) was used as an internal standard. Each sample was independently tested three times.

## Conclusions

In this study, an optimized method of embryo sac chromosome doubling using high temperatures in *P*. *simonii* × *P*. *nigra* var. *italica* was proposed, in which female catkins were exposed to 41 °C for 2 h at 66 HAP. Twelve polyploids were identified by a combined strategy of a chloroplast counting method and flow cytometry, and the rate of triploid induction was 23.41%, which was higher than that reported in a previous study by our group (11.76%)^14^. These results indicate an efficient process for chromosome doubling in *P*. *simonii* × *P*. *nigra* var. *italica*. A material system of polyploids (two parents and offspring groups with different ploidy levels) was constructed in this study and will be useful for *Populus* breeding and plant evolution studies.

## Supplementary information

Supplemental information
