# Supplementary material for: An improved method for high-temperature induced embryo sac chromosome doubling in Populus simonii Carr. × P. nigra var. italica (Moench.) Kochne, an interspecific hybrid of Tacamahaca and Aigeiros poplars
Source: Sci Rep. 2019 Jan 24;9:551. doi: 10.1038/s41598-018-37297-w (PMC6346037; doi:10.1038/s41598-018-37297-w)
Supplement: Supplementary file 1 — Supplemental information [file 41598_2018_37297_MOESM1_ESM.pdf]

An improved method for high-temperature induced embryo sac chromosome doubling in *Populus simonii* Carr. × *P. nigra* var. *italica* (Moench.) Kochne, an interspecific hybrid of *Tacamahaca* and *Aigeiros* poplars

Wenting Xu[1, 2#], Liqin Guo[1, 3#], Yan Zhang[1], Jian Zhao[1], Zunzheng Wei[4]*, Jinfeng Zhang[1]*

[1]Beijing Advanced Innovation Center for Tree Breeding by Molecular Design, National Engineering Laboratory for Tree Breeding, Key Laboratory of Genetics and Breeding in Forest Trees and Ornamental Plants of Ministry of Education, Key Laboratory of Forest Trees and Ornamental Plants biological engineering of State Forestry Administration, College of Biological Sciences and Technology, Beijing Forestry University, Beijing, 100083, P. R. China

[2]State Key Laboratory of Subtropical Silviculture, School of Forestry and Biotechnology, Zhejiang A&F University, Hangzhou, 311300, P. R. China

[3]Beijing Academy of Forestry and Pomology Sciences, Beijing, 100093, P. R. China

[4]Key Laboratory of Biology and Genetic Improvement of Horticultural Crops (North China), Ministry of Agriculture, Key Laboratory of Urban Agriculture (North), Ministry of Agriculture, Beijing Vegetable Research Center, Beijing Academy of Agriculture and Forestry Sciences, Beijing, 100091, P. R. China

*Corresponding Authors

Jinfeng Zhang: zjf@bjfu.edu.cn

Zunzheng Wei: weizunzheng@163.com

[#]These authors contributed equally to this work.

Supplementary table S1 Developmental process of megasporocytes after pollination in *P. simonii* × *P. nigra* var. *italica*

Time after pollination (h)	Numbers of embryo sac in each developmental stage							Total numbers
	Meiosis I	Meiosis II	Uni-nucleate embryo sac	Two-nucleate embryo sac	Four-nucleate embryo sac	Eight-nucleate embryo sac	Mature embryo sac and post	
0	8	18	23	9	5			63
12	3	20	21	11	4	1		60
24	2	22	24	13	6	1		68
36	2	12	23	16	7	3		63
48		10	20	21	9	4	2	66
60			18	23	15	7	4	67
72			11	22	12	13	9	67
96			3	12	13	17	14	59
120					11	27	23	61
144						21	32	53

Supplementary table S2 Survival capsules in high temperature preliminary experiment

Treatment duration(h)	temperature(℃)			
	36	41	46	CK
1	99/113	108/119	4/131	104/118
3	122/135	61/127	0/116	
5	88/102	0/133	0/124	
